# The Magnitude and Directions of the Associations between Early Life Factors and Metabolic Syndrome Differ across Geographical Locations among Migrant and Non-Migrant Ghanaians—The RODAM Study

**DOI:** 10.3390/ijerph182211996

**Published:** 2021-11-15

**Authors:** Thijs G. W. van der Heijden, Felix P. Chilunga, Karlijn A. C. Meeks, Juliet Addo, Ina Danquah, Erik J. Beune, Silver K. Bahendeka, Kerstin Klipstein-Grobusch, Frank P. Mockenhaupt, Mitzi M. Waltz, Charles Agyemang

**Affiliations:** 1 Department of Public and Occupational Health, Amsterdam Public Health Research Institute, Amsterdam University Medical Center, University of Amsterdam, 1105 AZ Amsterdam, The Netherlands; f.p.chilunga@amsterdamumc.nl (F.P.C.); e.j.beune@amsterdamumc.nl (E.J.B.); c.o.agyemang@amsterdamumc.nl (C.A.); 2 Center for Research on Genomics & Global Health, National Human Genome Research Institute, National Institutes of Health, Bethesda, MD 20894, USA; karlijn.meeks@nih.gov; 3 Department of Non-Communicable Disease Epidemiology, London School of Hygiene and Tropical Medicine, London WC1E 7HT, UK; juliet.x.addo@gsk.com; 4 Heidelberg Institute of Global Health (HIGH), Faculty of Medicine and University Hospital, Heidelberg University, 69120 Heidelberg, Germany; ina.danquah@uni-heidelberg.de; 5 Mother Kevin Postgraduate Medical School (MKPGMS), Uganda Martyrs University, Kampala 32297, Uganda; Bahendeka@yahoo.com; 6 Julius Global Health, Julius Center for Health Sciences and Primary Care, University Medical Centre, Utrecht University, 3584 CG Utrecht, The Netherlands; k.klipstein-grobusch@umcutrecht.nl; 7 Division of Epidemiology and Biostatistics, Department of Public Health, Faculty of Health Sciences, University of the Witwatersrand, Johannesburg 2193, South Africa; 8 Institute of Tropical Medicine and International Health, Charité-University Medicine Berlin, 13353 Berlin, Germany; frank.mockenhaupt@charite.de; 9 Athena Institute, Vrije Universiteit Amsterdam, 1081 HV Amsterdam, The Netherlands; m.m.waltz@vu.nl

**Keywords:** metabolic syndrome, migration, early life factors, obesity, diabetes

## Abstract

Background: Early-life factors (ELFs) such as childhood nutrition and childhood socio-economic status could be the drivers of the increase in metabolic syndrome (MetSyn) among African populations, but data are lacking. This study evaluated whether markers of childhood nutritional status and childhood socio-economic status were associated with MetSyn in adulthood among migrant Ghanaians living in Europe and non-migrant Ghanaians living in Ghana. Methods: Data from the Research on Obesity and Diabetes among African Migrants (RODAM) study, involving 2008 migrants and 2320 non-migrants aged ≥25 years, were analysed for this study. We used leg-length to height ratio (LHR), which is an anthropometric marker of childhood nutritional status, and parental education, which is a marker of childhood socio-economic status, as proxies. Adjusted odds ratios (AOR) and 95% confidence intervals (95% CI) were calculated by logistic regression with adjustments for demographic and lifestyle factors. Results: Parental education was higher among Ghanaians in Europe than among residents in rural and urban Ghana. The prevalence of MetSyn was 18.5%, 27.7% and 33.5% for rural, urban, and migrant residents, respectively. LHR was inversely associated with MetSyn among migrants. Compared with high paternal education, individuals with low paternal education had lower odds of MetSyn in migrants (AOR 0.71 95% CI 0.54–0.94). In contrast, compared with high maternal education, individuals with intermediate maternal education had higher odds of MetSyn in urban Ghanaians (AOR 4.53 95% CI 1.50–3.74). No associations were found among rural Ghanaians. Conclusion: The magnitude and direction of the associations between ELFs and MetSyn differ across geographical locations. Intermediate maternal education was positively associated with MetSyn among urban Ghanaians, while LHR and low paternal education were inversely associated with MetSyn among migrant Ghanaians. Further research into the interplay of genetics, environment and behaviour is needed to elucidate the underlying pathological mechanisms of MetSyn amongst migrants.

## 1. Introduction

Low and middle-income countries, including most sub-Saharan African countries, are experiencing epidemiological transitions as the burden of disease shifts from communicable towards non-communicable diseases, such as type 2 diabetes mellitus and cardiovascular diseases [1,2]. Metabolic syndrome (MetSyn) is defined by five cardio-metabolic risk factors, i.e., increased waist circumference, elevated triglycerides, elevated blood pressure, lowered high-density lipoprotein cholesterol, and elevated fasting plasma glucose, and is similarly increasing in the region [3]. MetSyn and its factors are associated with type 2 diabetes and cardiovascular disease morbidity and mortality [3].

The emergence of MetSyn among populations from sub-Sahara Africa is thought to be driven by economic development and urbanization, which is accompanied by shifts in diet and lifestyle [4]. For instance, urbanization has been shown to lead to diets high in fats and carbohydrates, increased physical inactivity, tobacco smoking and alcohol consumption [1]. These lifestyle and nutritional shifts are thought to drive the increasing gradient of MetSyn from rural Africa through urban Africa to African migrants residing in high-income countries [5]. However, lifestyle factors do not fully explain the rising prevalence of MetSyn among populations from sub-Saharan Africa [6].

Adverse early-life factors (ELFs) could potentially be another contributor to the rising burden of MetSyn among sub-Saharan African populations. Adverse ELFs, such as poor childhood nutrition status and low childhood socio-economic status, are highly prevalent in sub-Saharan Africa and could have a major influence on MetSyn later in adulthood [7,8]. Low birthweight, undernutrition and pre-term birth have been associated with increased risks of non-communicable diseases such as type 2 diabetes, obesity and cardiovascular diseases [9,10]. Childhood socio-economic status has an inverse association with risks for non-communicable diseases. Upward mobility, i.e., attaining a higher socio-economic status by education or occupation, only partly mitigates the effects of ELFs [11]. The explanation of the contribution of ELFs lies in the mismatch hypothesis of the developmental origins of health and disease theory. The developmental origins of health and disease theory recognizes the influence of environmental factors in developmental stages on the risk of acquiring non-communicable chronic diseases in later life [12]. The mismatch hypothesis states that a deprived environment in terms of nutrition during pregnancy and early childhood leads to a phenotype adapted to these deprived conditions, therefore economizing the energy metabolism and selecting for an obesogenic phenotype [12,13]. This obesogenic phenotype is a mismatch for the rich nutritional environment after urbanization or migration, linked to development of cardiometabolic health problems [14]. Despite the link between ELFs and an individual’s risk of non-communicable diseases in adulthood, data on how ELFs affect non-communicable diseases in urbanizing/globalizing populations are scant [15].

Therefore, we hypothesized that sub-Saharan Africans with lower childhood socio-economic status and higher prevalence of childhood undernutrition have an increased risk of acquiring MetSyn in an urban environment in Africa or following migration to high-income countries in later life [1,16]. Using the Research on Obesity and Diabetes among Migrants (RODAM) study data, we aimed to examine associations between proxies for ELFs and prevalence of MetSyn among adult migrant Ghanaians in Europe and non-migrant Ghanaians in Ghana. As such, the current study builds on previous RODAM analyses, which assessed associations between ELFs and type 2 diabetes, as well as between ELFs and estimated 10-year cardiovascular disease risk [17,18]. Since MetSyn is a risk factor for both type 2 diabetes and cardiovascular diseases, our study investigates the effects of ELFs on important intermediate risk factors (MetSyn) well before type 2 diabetes and cardiovascular diseases are apparent [3]. The knowledge generated by this study may be used to design interventions for the cardiometabolic effects of ELFs that take into account MetSyn as an important step. For instance, screening for MetSyn could be performed beginning in young adulthood among those with a history of ELFs in order to prevent type 2 diabetes and cardiovascular diseases.

## 2. Materials and Methods

The rationale, conceptual framework, design and methodology of the RODAM study have been previously published and will, therefore, be summarized here [16].

### 2.1. Study Design and Recruitment Procedures

The RODAM study is a multicenter cross-sectional study that was conducted between 2012 and 2015. The population was a homogenous group of sub-Saharan Africans of Ghanaian ancestry over 25 years old and living in Ghana (rural, urban) or in European cities (Amsterdam, Berlin and London). Participants were recruited using a random sampling procedure from municipality registration or Ghanaian organizations in the three European sites, or from rural and urban enumerations areas in the Ashanti region of Ghana. The participation rates were for rural Ghana 76%, urban Ghana 74%, London 75%, Amsterdam 53%, and Berlin 68%. Ethical approval was obtained in all the RODAM study sites. Informed written consent was obtained from each participant before enrolment in the RODAM study [16].

### 2.2. Eligibility

A total of 5659 out of 6385 participants were eligible for inclusion after written informed consent, completed questionnaires and physical examinations. After exclusion of individuals under 25 years and above 70 years of age and missing data, 4328 participants were available for subsequent analyses.

### 2.3. Data Collection

Data on the socioeconomic status of parents, migration-related factors, health status and behaviour, demographics, physical activity and current treatment/medication were obtained using structured questionnaires that were either self-administered or administered via face-to-face interviews conducted by trained ethnically matched research assistants.

All anthropometric measurements were taken twice during physical examination by trained study personnel. The mean of the two measurements was used for analysis. Measurements for weight and height were performed with validated devices (SECA877 weighing scale, SECA217 stadiometer) in light clothing without shoes. Leg length, trunk length, and leg-length to height ratio (LHR) were calculated from standing and sitting height measurements. Hip and waist circumference (defined as midpoint between the lower rib and upper margin of the iliac crest) were measured using a tape measure. The waist to hip ratio was calculated to determine abdominal obesity using the World Health Organization criteria.

Systolic and diastolic blood pressure were measured three times after at least 5 minutes of rest in a sitting position using the Microlife WatchBP Home (Veenendaal, The Netherlands: Microlife Benelux.) For analyses, the mean of the last two measurements was used. Fasting venous blood samples were drawn from participants by trained research assistants. Standardized pre-analytic procedures and shipping were used across all locations. Fasting glucose plasma, HDL-cholesterol, and triglycerides levels were quantified using the ABX Pentra 400 chemistry analyser (Montpellier, France: HORIBA ABX).

### 2.4. Metabolic Syndrome (MetSyn)

Participants were categorized as having metabolic syndrome (MetSyn) based on the harmonized criteria if they had three of the following five conditions: raised fasting glucose, raised blood pressure, elevated triglycerides, reduced HDL-cholesterol, and increased waist circumference (Table 1) [3].

### 2.5. Early-Life Factors (ELFs)

Leg length and trunk length are widely used indicators for nutritional status, as they reflect lifestyle and nutritional influences during infancy, childhood and the onset of puberty [19,20,21]. The growth of legs happens earlier and faster than growth of the trunk, and contributes to the majority of variability in height [19,22]. Therefore, the ratio of leg-length and height is an indicator of growth in early childhood [18,19,23,24,25]. Leg-length to height ratio (LHR) was hence used as a proxy for childhood nutritional status, as growth is nutrition dependent [18,23,24,25]. We used parental education as a proxy for socio-economic status during childhood, since education has been positively correlated with income in sub-Saharan Africa [8]. When parents had no or elementary education, it was categorized as low parental education, lower secondary/vocational as intermediate parental education, and higher vocational/secondary and university as higher parental education.

### 2.6. Data Analysis

Data analyses were performed using Stata 16 (College Station, Texas, USA: StataCorp LLC; sourced from Vrije University Amsterdam). Descriptive statistics were presented as percentages, means, or as median and inter-quartile ranges when the distribution was skewed. Differences between baseline characteristics were assessed via ANOVA or the Kruskal–Wallis test (when skewed) for continuous variables and by chi-square tests for categorical variables.

Logistic regression analysis was performed to assess the association between ELFs maternal educational level and paternal educational level and MetSyn per location (outcome variable). Additionally, a logistic regression was performed for the association between LHR and MetSyn as a continuous variable because it was normally distributed (Appendix A).

Four logistic regression models were constructed to analyze the data. Model 0 was crude (non-adjusted). Model 1 adjusted for sex, age, own education and, for females’ age at menarche. We adjusted for age at menarche because it has been indicated to create differentials for height, fat mass and cardiovascular disease risks, and thus for MetSyn, between males and females [26,27]. Model 2 adjusted for Model 1 plus alcohol consumption, smoking, and physical activity and energy intake. Model 3 adjusted for Model 2 plus length of stay (migrants). Additionally, we stratified the analyses by cities in Europe for the migrant group (Amsterdam, Berlin and London).

## 3. Results

### 3.1. Study Population

In Table 2, the baseline characteristics of the study population are presented, stratified by geographical location. The analyses included: 2008 migrants, 1379 urban Ghanaians and 941 rural Ghanaians. A total of 71.8% of urban Ghanaians were female compared to 61.1% of rural Ghanaians and 57.9% of migrants. The average age among migrants was higher (47.0 ± 9.7 years) compared to rural Ghanaians (46.3 ± 12.6 years) and urban Ghanaians (45.3 ± 11.5 years).

Educational levels followed a gradient from lowest in rural Ghana through urban Ghana to highest in migrants. The mean LHR was ranged from 0.499 (±0.02) in migrants to 0.51 (±0.02) in rural Ghanaians and 0.50 (±0.02) in urban Ghanaians. Migrant Ghanaians were the least active, were more likely to smoke and drink alcohol, and had a higher total energy intake compared to rural and urban Ghanaians.

### 3.2. Proportions of MetSyn by Context across ELFs

**Figure 1:**
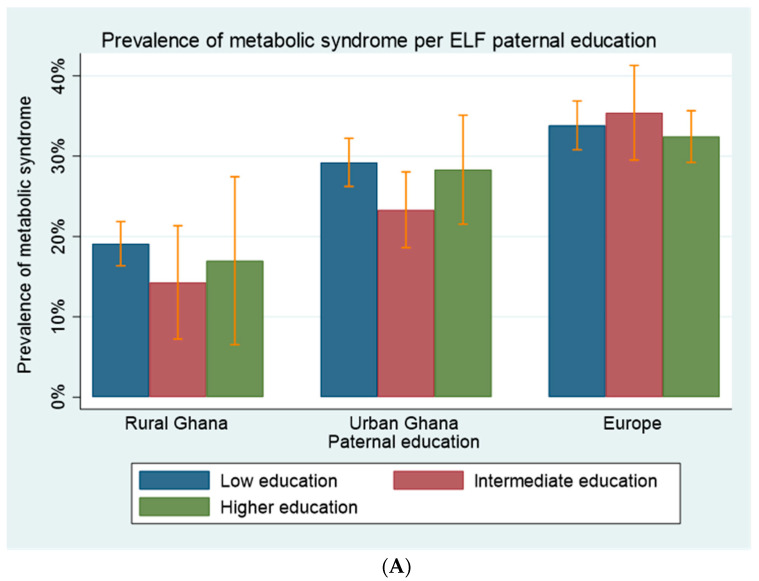

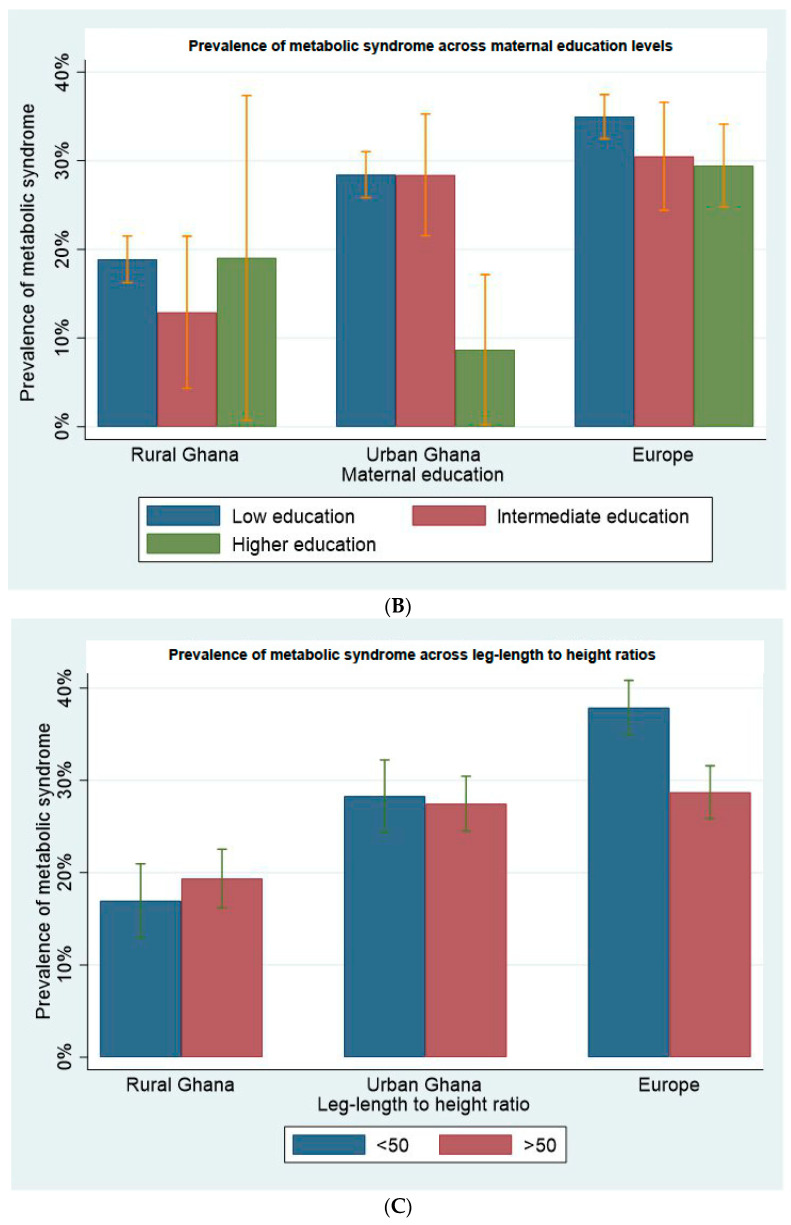
(**A**) Prevalence of metabolic syndrome across paternal education levels in rural Ghana, urban Ghana and among Ghanaian migrants in Europe. Error bars are the 95% confidence intervals. (**B**) Prevalence of metabolic syndrome across maternal education levels in rural Ghana, urban Ghana and among Ghanaian migrants in Europe. Error bars are the 95% confidence intervals. (**C**) Prevalence of metabolic syndrome across maternal education levels in rural Ghana, urban Ghana and among Ghanaian migrants in Europe. Error bars are the 95% confidence intervals.

The overall proportions of MetSyn in the analyzed population was 28.4%. The prevalence of MetSyn was 18.5% in rural Ghana, 27.8% in urban Ghana and 33.5% in migrants (Table 2, *p* < 0.001). The prevalence of MetSyn between those with high and low paternal and maternal education levels varied (Figure 1A,B). The prevalence of MetSyn was higher in those with lower LHR in Europe compared to those with higher LHR, while there was no difference between lower and higher LHR in urban and rural Ghana (Figure 1C).

### 3.3. Associations between ELFs and MetSyn

Associations between ELFs and MetSyn differed across the geographical locations (Table 3). No associations between ELFs and MetSyn were detected among rural Ghanaian residents. Higher LHR means that a person has longer legs and is unlikely to have suffered from malnutrition [19].

LHR was inversely associated with MetSyn among migrants in all models, but only significant in models 0 through 2. LHR was not associated with MetSyn among rural or urban Ghanaians. Paternal education was associated with MetSyn in migrants, and maternal education in urban Ghanaians. Compared to migrants with high paternal education, migrants with low paternal education had lower odds of MetSyn after adjustments for all covariates (AOR 0.71, 95% CI 0.54–0.94). Urban Ghanaians with intermediate maternal education had higher odds of MetSyn after adjustment for all covariates compared to those with higher maternal education (AOR 4.53, 95% CI 1.50–3.74).

### 3.4. Stratified Analysis by Site in Europe

Stratified analysis by site in Europe found that the prevalence of MetSyn was 36.3% in Amsterdam, while it was 23.1% and 35.6% respectively for the Berlin and London Ghanaians. The trends of MetSyn for paternal, maternal education and LHR were similar for Amsterdam and London Ghanaians, but differed to those of Berlin Ghanaians (Appendix B). In the logistic regression models, associations of low parental education with MetSyn were not apparent. The association between higher LHR and a reduced risk on MetSyn was only seen in Amsterdam (AOR 0.86, 95% CI 0.73–0.99) after adjusting for covariates (Appendix C).

## 4. Discussion

In the present study, we found that LHR was inversely associated with MetSyn among migrants, and that migrants with low paternal education have lower odds of MetSyn compared with migrants with higher paternal education. In urban Ghana, participants with intermediate maternal education were observed to have higher odds of MetSyn compared to participants with higher maternal education. We found no associations between LHR or parental education with MetSyn among rural Ghanaians.

The pathway from normal health to cardiometabolic diseases such as type 2 diabetes and cardiovascular diseases involves multiple steps (pathways). One important intermediate step is the development of MetSyn, where a constellation of cardiometabolic factors (increased waist circumference, elevated triglycerides levels, elevated blood pressure, lowered HDL-cholesterol, and elevated fasting blood glucose levels) develop well before type 2 diabetes and cardiovascular diseases are apparent [3]. Since ELFs have been associated with type 2 diabetes and cardiovascular diseases in various population groups (including urbanizing/globalizing Ghanaians) [17,18], the intermediate step of MetSyn might provide an important tool in the prevention of cardiometabolic diseases. For instance, all children with history of ELFs could be periodically screened for MetSyn in adulthood in order to prevent type 2 diabetes and cardiovascular diseases. As such, we had hypothesized that sub-Saharan African individuals with lower childhood SES and higher prevalence of childhood undernutrition would have higher odds of acquiring MetSyn in an urban environment in Africa or following migration to high-income countries in later life.

In the current analyses, we found an inverse relationship between LHR and MetSyn in migrants. Based on previous RODAM studies where LHR was associated with waist circumference and with type 2 diabetes, this finding was expected, as a higher LHR may equate to better early life nutrition, which in turn might reduce the odds of MetSyn in adulthood [18]. Previous studies have shown that LHR is correlated with childhood nutrition status, whereby children with better nutrition have higher LHR [19,21,28]. Therefore, LHR serves as a proxy for early life nutrition status. The inverse relationship between LHR and MetSyn is consistent with previous studies from high-income countries where malnutrition during childhood was associated with higher rates of cardiometabolic diseases in adulthood [19,21,28,29,30]. At present, it remains unclear what the exact pathogenic processes are but there are some possible postulations.

First, maternal undernutrition during pregnancy can lead to MetSyn in adulthood via fetal programming, whereby a lack of nutrients for fetal metabolism leads to epigenetic maladaptations. This can give rise to low birth weight, an “economized metabolism,” and changes in growth during childhood. An economized metabolism can predispose individuals to MetSyn through hormone imbalances, increased adipose tissue and inflammation [1,12,31]. Second, nutritional status in childhood influences individual phenotype, as long-term exposure to malnutrition (either under- or over-nutrition) selects for phenotypic traits that lead to cardiometabolic diseases [12,14]. This process is described as the plasticity of the relationship between phenotype and genotype, whereby the ability to change the phenotype decreases as the person gets older [14]. Third, mismatch between the developmental environment (fetal and childhood) and the environment in later life further exacerbates the positive selection for cardiometabolic diseases (mismatch hypothesis) [32]. If the diet in adulthood is different to the diet in childhood, the body’s metabolic machinery is not well-equipped to deal with the change, resulting in poor metabolism and eventually MetSyn [12]. For example, childhood malnutrition can lead to an insulin resistance phenotype in individuals from a rural Ghanaian background. If this person migrates to a high-income country in later life, the new high-income country diet is a mismatch with their developmental background, resulting in a higher fasting blood glucose level. We postulate that similar processes could have occurred in our study population. Ghana has experienced repeated drought periods that have been linked to stunting and lower LHR, such as the major drought of 1981–1983 [33,34]. Over 80% of our participants could have been exposed to these periods of drought, and resulting famine and malnutrition. The combination of early exposure to famine during childhood and adoption of unhealthy behaviors in later life could explain why LHR is inversely associated with MetSyn among migrants. The adoption of unhealthy behaviors upon migration leading to MetSyn is further evidenced by the increase in non-alcoholic fatty liver disease, which is the hepatic manifestation of MetSyn and also results from unhealthy lifestyle factors [35,36].

It should also be noted that the effect of LHR on MetSyn was not apparent in Ghana, and was attenuated after adjusting for length of stay in Europe among migrants. This shows that there is indeed an interaction between early life nutrition (as measured by LHR) and later change in the wider environment in adulthood (length of stay in Europe upon migration). One example could be that of chronic infections. Chronic infections such as worms and malaria can increase the risk of MetSyn via chronic inflammatory pathways [37,38]. These chronic infections are more common in Ghana and are relatively less common in Europe. Therefore, having an LHR >0.50 (better early life nutrition) in Ghana may not substantially reduce the risk of MetSyn in Ghana due to the concurrent presence of infections that also increase the risk of MetSyn, while in Europe an LHR >0.50 (better early life nutrition) combined with a lack of chronic infections may largely reduce the risk of MetSyn. Since we could not explore the role of many unmeasured factors (e.g., chronic infections, psychosocial stress etc.) in our regression models, further studies should explore how these unmeasured factors mediate the relationship between early life factors and MetSyn [39].

We found that migrants whose fathers have low education had a lower rate of MetSyn than migrants whose fathers were highly educated. We used parental education as a proxy for higher socio-economic status during childhood, since education has been positively correlated with income in sub-Saharan Africa [40,41,42,43]. Low socio-economic status is associated with poor health, especially in high income countries, where low childhood socio-economic status is associated with cardiometabolic diseases and other poor health outcomes [11,40,44]. Our finding is in direct contrast with findings amongst European populations [11,40,45]. Low socio-economic status might have a double-edged effect, as low socio-economic status in adulthood (possibly not in children) in Africa is protective against MetSyn due to lack of access to fast food and more physical activity. However, if it happens in childhood, it increases MetSyn due to malnutrition and poor birth outcomes. For persons with high parental socio-economic status, it might be that parents were poor when the child was born, resulting in suffering from both poor early life factors and excess nutrition due to improved parental socio-economic status (socio-economic status is longitudinal and can change over time), resulting in a larger mismatch. The exact explanation for the association between low paternal education and MetSyn in this study is unclear and requires further investigation. However, migration history and lifestyle factors might have more influence on MetSyn than ELFs (like childhood nutrition status or childhood socio-economic status) due to their more proximate position to the onset of the disease.

This study found that there was an inverse gradient in the association between maternal education and MetSyn in urban Ghana where low and intermediate education were associated with MetSyn, although the former was non-statistically significant. Nonetheless, maternal education, also a proxy for childhood socio-economic status in this study, is positively associated with earlier preventive care initiation, according to previous research [46]. It might be possible that maternal education is more important than paternal education for a healthy lifestyle and thus for the risk of MetSyn in this study. This gradient follows the hypothesis that low socio-economic status in childhood results in more risk of MetSyn in later life. Due to low maternal education, a child can be exposed to malnutrition, fetal undernutrition and low birth weight [1,42,43,47]. Exposure to these factors in early life can possibly exacerbate the risk of cardiometabolic diseases in urban contexts where there is a shift to unhealthy diets, sedentary lifestyles and smoking in adulthood [42]. However, the effects observed in this study could also be influenced by experiences in young adulthood, as socio-economic status changes over time. These results could be the result of a balance between early life and young adulthood factors that all influence MetSyn in later life.

Among migrants, we found negative associations between LHR and MetSyn in Amsterdam as opposed to Berlin and London. The observed differences in Amsterdam, Berlin and London could be attributed to contextual factors such as access to health care and preventative services, diet and psychosocial factors, which differ between the cities and may play a role in the pathogenesis of MetSyn. Additionally, the rural or urban origin of a migrant could play a role, as the change in lifestyle factors is greater for migrants from rural areas than those from urban areas. This change in lifestyle factors combined with poorer early life nutrition (as measured by LHR) in rural areas could lead to the observed differences. However, this is unlikely, as all the European migrant groups originated both from rural and urban sites in Ghana.

### Strengths and Limitations

The main strength of the study is the inclusion of a large homogenous group of Ghanaian adults over five geographical locations. As the participants were first generation migrants, it is likely that all participants were exposed to similar childhood social circumstances, as they all experienced their childhood in Ghana. The study has some limitations as proxies were used for ELFs. LHR can be affected by SES and genetic components, yet it is a widely accepted proxy for childhood nutritional status [19,21]. The second limitation is that there is no known cut-off for defining malnutrition for the LHR as an ELF, as there is no population data available to derive this cut-off from. However, LHR was normally distributed in our study population, hence our logistic regression results clearly show that migrants with the lowest LHR (lowermost extreme) have higher odds of MetSyn than those with the highest LHR (uppermost extreme). This is an important finding, despite the lack of a defined cut-offs for LHR in categorizing those with malnutrition. A third limitation is that the quantification of childhood SES is a multifactorial sum that is not solely explained by a one-dimensional proxy like parental education [41]. Also, upward mobility of parents’ SES and influences from extended families raising children together cannot be ruled out, and can thus alter childhood SES [48]. However, using a proxy like parental education is the most reliable quantification due to the lowest chance of recall bias [41]. Another issue is that paternal education can be a biased indicator, as we do not know if the father was present growing up, or whether he was a biological father or stepfather. Finally, the cross-sectional nature of this study precludes determining any causality.

## 5. Conclusions

The magnitude and direction of the associations between ELFs and MetSyn differed largely across the geographical locations. No associations between ELFs and MetSyn were found among rural Ghanaians. This study found that among migrants, LHR and low paternal education were inversely associated with MetSyn, while intermediate maternal education was positively associated with MetSyn among urban Ghanaians. These results provide insight into how ELFs such as childhood malnutrition and SES relate to MetSyn in adulthood in populations undergoing epidemiological transitions. The results of this research indicate that individuals in the migrant population at risk for MetSyn before onset of the disease could be identified by use of ELFs. Nonetheless, that requires further longitudinal studies to identify context-specific pathological and behavioral mechanisms that underlie associations between ELFs and MetSyn.

## Figures and Tables

**Table 1:** Harmonized definition of metabolic syndrome (adapted from Alberti et al., 2009).

Risk Factor	Participant Has Risk Factor Yes/No
Elevated triglycerides	≥150 mg/dL (1.7 mmol/L) or receiving treatment for risk factor
Reduced high-density lipid cholesterol	<40 mg/dL (1.0 mmol/L) in males; <50 mg/dL (1.3 mmol/L) in females or receiving treatment for risk factor
Elevated blood pressure	Systolic ≥ 130 and/or diastolic ≥ 85 mmHg or receiving treatment for risk factor
Elevated fasting glucose plasma	≥100 mg/dL or receiving treatment for risk factor
Increased waist circumference for Sub-Saharan Africans ^1^	Males ≥ 94 cm; Females ≥ 80 cm

^1^ This criterion is ancestry/ethnicity dependent, so for this study only the relevant increased waist circumference cut off for African ancestry is used.

**Table 2:** Baseline characteristics of participants included in present analyses.

	Rural Ghanaians	Urban Ghanaians	Migrant Ghanaians	*p*-Value ^6^
Numbers enrolled, n (%)	941 (21.74)	1379 (31.86)	2008 (46.39)	
Women, n (%)	575 (61.10)	991 (71.86)	1162 (57.87)	<0.001
Age in years (mean ± SD)	46.3 ± 12.6	45.3 ± 11.5	47.0 ± 9.7	<0.001
Education, n (%)				<0.001
Never/elementary only	539 (57.28%)	603 (43.73)	434 (21.56)	
Lower vocational/secondary school	296 (31.35)	538 (39.01)	750 (36.95)	
Higher vocational/secondary school	72 (7.55)	172 (12.47)	479 (23.75)	
University	35 (3.72)	65 (4.71)	341 (16.88)	
Metabolic syndrome, n (%)	174 (18.49)	383 (27.77)	672 (33.47)	<0.001
Systolic Blood Pressure, mmHg (median (IQR))	119.5 (110–133.5)	123.5 (112.5–136)	132.5 (122.75–144.5)	<0.001
Hypertensive, n (%)	275 (29.22)	506 (36.69)	1.161 (57.82)	<0.001
BP medication, n (%) ^1^	127 (13.50)	263 (19.07)	695 (34.61)	<0.001
Total cholesterol, mmol/L (mean ± SD)	4.50 ± 1.13	5.21 ± 1.15	5.07 ± 1.07	<0.001
LDL cholesterol, mmol/L (mean ± SD) ^2^	2.79 ± 0.95	3.43 ± 0.99	3.23 ± 0.94	<0.001
HDLcholesterol, mmol/L (mean ± SD) ^3^	1.20 ± 0.38	1.26 ± 0.32	1.42 ± 0.34	<0.001
Cholesterol medication, n (%)	17 (1.81)	26 (1.89)	277 (13.79)	<0.001
Median tryglycerides, mmol/L	0.98 (0.74–11.31)	1.02 (0.74–1.35)	0.79 (0.61–1.05)	<0.001
Blood glucose, mmol/L	4.91 (4.59–5.30)	5.1 (4.77–5.46)	5.09 (4.71–5.62)	<0.001
Diabetes medication, n (%)	23 (2.44)	73 (5.29)	164 (8.17)	<0.001
Waist circumference,(mean ± SD)	81.11 ± 10.76	89.26 ± 11.74	94.76 ± 11.66	<0.001
Abdominal obesity n (%) ^4^	184 (19.55)	582 (42.20)	1026 (51.10)	<0.001
Height (mean ± SD)	162.11 ± 8.57	161.74 ± 7.94	165.62 ± 7.99	<0.001
Leg length, (mean ± SD)	82.17 ± 5.47	81.65 ± 5.48	82.81 ± 5.52	<0.001
Leg-length to height ratio (LLHR), (mean ± SD)	0.51 ± 0.02	0.50 ± 0.02	0.50 ± -0.02	<0.001
Maternal Education, n (%)				<0.001
Lower education	858 (91.18)	1164 (84.40)	1415 (70.47)	
Intermediate education	62 (6.59)	169 (12.26)	223 (11.11)	
Higher education	21 (2.23)	46 (3.34)	370 (18.42)	
Paternal Education, n (%)				<0.001
Lower education	790 (83.95)	893 (64.76)	934 (46.51)	
Intermediate education	98 (10.41)	313 (22.69)	257 (12.80)	
Higher education	53 (5.63)	173 (12.55)	817 (40.69)	
Type 2 diabetes mellitus, n (%)	49 (5.21)	130 (9.43)	235 (11.70)	<0.001
Length of stay, (median (IQR))			16.38 (9.82–23.79)	N/A
Age of menarche (Female only), (mean ± SD)	14.86 ± 1.95	14.83 ± 1.55	14.64 ± 1.81	<0.001
Smoking, n(%)				<0.001
Current smoker	20 (2.13)	14 (1.02)	72 (3.59)	
Former smoker	62 (6.59)	82 (5.95)	150 (7.47)	
Alcohol-consumption, n(%)				<0.001
No alcohol	548 (58.24)	955 (69.25)	1134 (56.47)	
Alcohol	393 (41.76)	424 (30.75)	874 (43.53)	
Total energy consumption per day (median (IQR))	2588.3 (2055.5–3374.6)	2229.1 (1842.8–2697.3)	2600.6 (1946.6–3520.3)	<0.001
Physical Activity levels, n (%)				<0.001
Unknown	4 (0.43)	12 (0.87)	137 (6.82)	
Low	173 (18.38)	485 (35.17)	530 (26.39)	
Moderate	196 (20.83)	229 (16.61)	419 (20.87)	
High	568 (60.36)	653 (47.35)	922 (45.92)	
BMI, n (%) ^5^				<0.001
<25 kg/m^2^	715 (75.98)	555 (40.28)	414 (20.63)	
25–30 kg/m^2^	176 (18.70)	467 (33.89)	856 (42.65)	
≥30 kg/m^2^	50 (5.31)	356 (25.83)	737 (36.72)	

Statistical significance at *p* < 0.05, tests used one-way analysis of variance (ANOVA) to test for means, Kruskal–Wallis for medians and chi square test for frequencies (SD = standard deviation, IQR = interquartile range); ^1^ BP = blood pressure, ^2^ LDL = low-density lipid, ^3^ HDL = high-density lipid, ^4^ WHO = World Health Organization, ^5^ = body mass index. ^6^ *p*-value for the difference between groups obtained via chi square tests for categorical variables and ANOVA or Kruskal–Wallis tests (when skewed) for continuous variables mass index.

**Table 3:** Associations between early life factors and metabolic syndrome in the Research on Obesity and Diabetes among African Migrants (RODAM) study population (N = 4328) in the migrated Ghanaians, rural Ghanaians and urban Ghanaians.

Variable	Rural Ghanaians, OR (95% CI)					Urban Ghanaians, OR (95% CI)					Migrant Ghanaians, OR (95% CI)					
	n (%)	% MetSyn	Model 0 ^a^	Model 1 ^b^	Model 2 ^c^	n (%)	% MetSyn	Model 0 ^a^	Model 1 ^b^	Model 2 ^c^	n (%)	% MetSyn	Model 0 ^a^	Model 1 ^b^	Model 2 ^c^	Model 3 ^d^
Paternal education																
Lower educataion	790 (83.95)	19.11	1.16 (0.55–2.42)	0.61 (0.26–1.41)	0.57 (0.25–1.33)	893 (64.76)	29.22	1.05 (0.73–1.50)	0.75 (0.50–1.12)	0.77 (0.51–1.16)	934 (46.51)	33.83	1.07 (0.87–1.30)	0.76 (0.60–0.96)	0.69 (0.53–0.90)	0.71 (0.54–0.94)
Intermediate education	98 (10.41)	14.29	0.81 (0.33–2.03)	0.69 (0.26–1.82)	0.69 (0.26–1.85)	313 (22.69)	23.32	0.77 (0.50–1.17)	0.69 (0.44–1.08)	0.72 (0.46–1.13)	257 (12.80)	35.41	1.14 (0.85–1.53)	0.97 (0.71–1.33)	1.02 (0.70–1.47)	0.96 (0.66–1.47)
Higher eduaction	53 (5.63)	16.98	1.00 (ref)	1.00 (ref)	1.00 (ref)	173 (12.55)	28.32	1.00 (ref)	1.00 (ref)	1.00 (ref)	817 (40.69)	32.44	1.00 (ref)	1.00 (ref)	1.00 (ref)	1.00 (ref)
Maternal education																
Lower educataion	858 (91.18)	18.88	0.99 (0.33–2.98)	0.55 (0.16–1.91)	0.53 (0.15–1.88)	1164 (84.40)	28.43	4.17 (1.48–11.72)	2.67 (0.92–7.78)	2.70 (0.93–7.86)	1415 (70.47)	34.98	1.29 (1.00–1.65)	0.79 (0.59–1.04)	0.77 (0.55–1.06)	0.79 (0.56–1.11)
Intermediate education	62 (6.59)	12.90	0.63 (0.17–2.35)	0.61 (0.15–2.56)	0.63 (0.15–1.68)	169 (12.26)	28.40	4.16 (1.42–12.25)	4.49 (1.49–13.54)	4.53 (1.50–3.74)	223 (11.11)	30.49	1.05 (0.73–1.51)	0.94 (0.64–1.39)	0.91 (0.58–1.43)	0.96 (0.60–1.53)
Higher eduaction	21 (2.23)	19.05	1.00 (ref)	1.00 (ref)	1.00 (ref)	46 (3.34)	8.70	1.00 (ref)	1.00 (ref)	1.00 (ref)	370 (18.42)	29.46	1.00 (ref)	1.00 (ref)	1.00 (ref)	1.00 (ref)
Leg-Length to Height ratio (standardized values)																
Leg-Length to Height ratio	941 (100)	18.49	1.12 (0.95–1.31)	0.99 (0.84–1.18)	1.00 (0.84–1.19)	1379 (100)	27.77	0.96 (0.85–1.08)	0.92 (0.80–1.05)	0.92 (0.80–1.06)	2008 (100)	33.47	0.87 (0.79–0.96)	0.83 (0.75–0.92)	0.88 (0.78–0.99)	0.89 (0.79–1.01)

^a^ Model 0, crude odds ratios; ^b^ Model 1, adjusted for age, sex, education and age of menarche; ^c^ Model 2, adjusted for age, sex, education, age of menarche, smoking, activity level, daily alcohol consumption, energy intake; ^d^ Model 3, adjusted for age, sex, education, age of menarche, smoking, activity level, daily alcohol consumption, energy intake and length of stay; Abbreviations: CI, confidence interval; OR, odds ratio; N, sample size. Ref = reference group.

**Figure A1:**
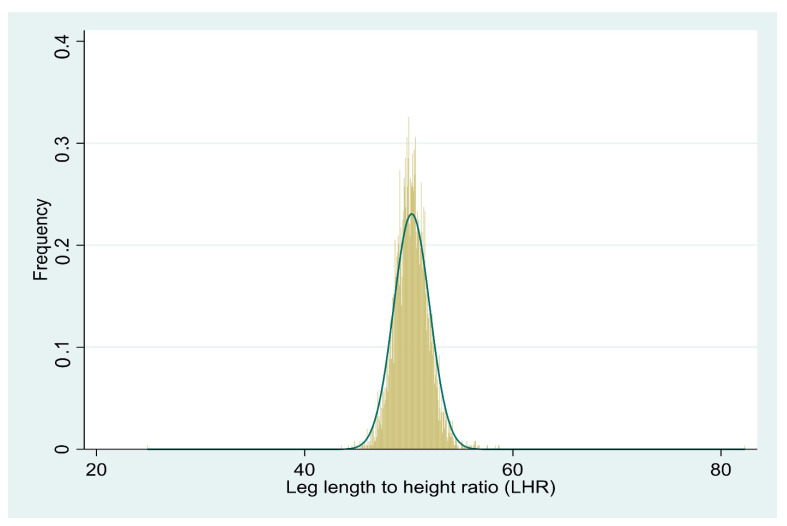
Distribution Plot of the Continuous Variable Leg-Length to Height Ratio (LHR) with a Normal-Density Curve.

**Figure A2:**
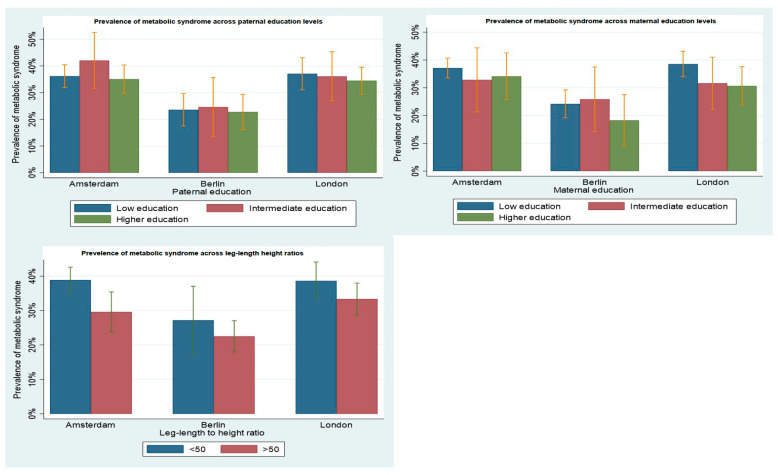
Prevalence of Metabolic Syndrome across Paternal Education, Maternal Education and Leg-Length to Height Ratios in Amsterdam, Berlin and London. Error Bars Are the 95% Confidence Interval.

## Data Availability

The RODAM data is available upon reasonable request from CA.

## Abbreviations

ELFs	Early life factors
MetSyn	Metabolic syndrome
RODAM	Research on Obesity and Diabetes Among Migrants
HDL-cholesterol	High-density lipoprotein cholesterol
LDL-Cholesterol	Low-density lipoprotein cholesterol
AOR	Adjusted odds ratio

## Appendix C

**Table A1:** Associations between Early Life Factors and Metabolic Syndrome among Migrant Ghanaians Stratified by Site in Europe.

Variable	Amsterdam Ghanaians, OR (95% CI)					Berlin Ghanaians, OR(95% CI)					London Ghanaians, OR(95% CI)				
	n (%)	% MetSyn	Model 0 ^a^	Model 1 ^b^	Model 2 ^c^	n (%)	% MetSyn	Model 0 ^a^	Model 1 ^b^	Model 2 ^c^	n (%)	% MetSyn	Model 0 ^a^	Model 1 ^b^	Model 2 ^c^
Paternal education															
Lower educataion	492 (55.22)	36.17	1.05 (0.78–1.41)	0.88 (0.63–1.24)	0.93 (0.66–1.32)	191 (46.59)	23.56	1.04 (0.63–1.72)	0.60 (0.33–1.06)	0.59 (0.33–1.06)	251 (35.50)	37.05	1.12 (0.80–1.57)	0.70 (0.47–1.03)	0.67 (0.44–1.00)
Intermediate education	88 (9.88)	42.05	1.34 (0.83–2.18)	1.40 (0.84–2.35)	1.22 (0.72–2.10)	61 (14.88)	24.59	1.11 (0.55–2.21)	0.90 (0.43–1.87)	0.89 (0.43–1.85)	108 (15.28)	36.11	1.07 (0.68–1.69)	0.79 (0.48–1.29)	0.74 (0.45–1.25)
Higher eduaction	311 (34.90)	35.05	1.00 (ref)	1.00 (ref)	1.00 (ref)	158 (38.53)	22.78	1.00 (ref)	1.00 (ref)	1.00 (ref)	348 (49.22)	34.48	1.00 (ref)	1.00 (ref)	1.00 (ref)
Maternal education															
Lower educataion	698 (78.34)	37.11	1.14 (0.76–1.70)	0.76 (0.48–1.19)	0.78 (0.49–1.24)	281 (68.54)	24.20	1.42 (0.74–2.76)	0.94 (0.45–1.95)	0.97 (0.47–2.01)	436 (61.67)	38.53	1.42 (0.97–2.06)	0.79 (0.51–1.22)	0.80 (0.50–1.28)
Intermediate education	67 (7.52)	32.84	0.94 (0.50–1.77)	0.84 (0.43–1.64)	0.82 (0.41–1.63)	58 (14.15)	25.86	1.55 (0.67–3.61)	1.74 (0.71–4.27)	1.80 (0.73–4.43)	98 (13.86)	31.63	1.05 (0.61–1.79)	0.87 (0.49–1.55)	0.94 (0.52–1.70)
Higher eduaction	126 (14.14)	34.13	1.00 (ref)	1.00 (ref)	1.00 (ref)	71 (17.31)	18.31	1.00 (ref)	1.00 (ref)	1.00 (ref)	173 (24.47)	30.63	1.00 (ref)	1.00 (ref)	1.00 (ref)
Leg-Length to Height ratio (standardized values)															
Leg-Length to Height ratio	891 (100)	36.36	0.88 (0.74–1.04)	0.81 (0.67–0.97)	0.86 (0.73–0.99)	410 (100)	23.41	1.00 (0.77–1.28)	0.94 (0.72–1.24)	0.95 (0.74–1.22)	707 (100)	35.64	0.99 (0.84–1.16)	0.91 (0.76–1.09)	0.96 (0.81–1.15)

^a^ Model 0, crude odds ratios; ^b^ Model 1, adjusted for age, sex, education and age of menarche; ^c^ Model 2, adjusted for age, sex, education, age of menarche, smoking, activity level, daily alcohol consumption, energy intake and length of stay; Abbreviations: CI, confidence interval; OR, odds ratio; N, sample size. Ref = reference group.
